# Cardiac computed tomography–derived fat attenuation index in coronary vasculitis: inflammation development over a year

**DOI:** 10.1093/ehjcr/ytaf532

**Published:** 2025-11-22

**Authors:** Leonie M Becker, Joyce Peper, Jan-Willem Balder, Jurriën M ten Berg, Martin J Swaans

**Affiliations:** Department of Cardiology, St. Antonius Hospital, Koekoekslaan 1, 3435 CM Nieuwegein, The Netherlands; Department of Radiology, University Medical Center Utrecht, Heidelberglaan 100, 3584 CX Utrecht, The Netherlands; Department of Cardiology, St. Antonius Hospital, Koekoekslaan 1, 3435 CM Nieuwegein, The Netherlands; Department of Cardiology, St. Antonius Hospital, Koekoekslaan 1, 3435 CM Nieuwegein, The Netherlands; Department of Cardiology, University Medical Center Utrecht, Heidelberglaan 100, 3584 CX Utrecht, The Netherlands; Department of Cardiology, St. Antonius Hospital, Koekoekslaan 1, 3435 CM Nieuwegein, The Netherlands; Cardiovascular Research Institute Maastricht (CARIM), Universiteitssingel 40, 6229 ER Maastricht, The Netherlands; Department of Cardiology, St. Antonius Hospital, Koekoekslaan 1, 3435 CM Nieuwegein, The Netherlands

## Case description

Routine imaging techniques have inherent limitations and often fail to detect coronary vasculitis until cardiovascular disease becomes manifest.^1^ In atherosclerosis, inflammatory activity precedes morphological changes and increases pericoronary fat attenuation on computed tomography (CT).^2^ The fat attenuation index (FAI, *CaRi Heart Analysis, Caristo Diagnostics*) may therefore support vasculitis assessment.

We evaluated a 62-year-old man with unexplained fever and recent stenting of the left anterior descending artery (LAD) for stable angina. When angina recurred, invasive coronary angiography (ICA) revealed new narrowing of the previously normal left circumflex (LCx), initially attributed to spasm. Soon after, polyarteritis nodosa was diagnosed. Before immunosuppressive therapy could be initiated, the patient experienced a myocardial infarction (MI). Coronary CT angiography (CCTA) and ICA showed in-stent occlusion and severe LCx compression due to coronary vasculitis. The patient subsequently underwent coronary bypass grafting (CABG).

Follow-up positron emission tomography (PET) showed unchanged isolated LAD activity, while CCTA showed reduced pericoronary fat induration at 3 months after CABG, but new induration around the right coronary artery (RCA) at 6 months, coinciding with persistently elevated inflammatory markers. At 12 months, induration on CCTA remained unchanged.

**Figure 1:**
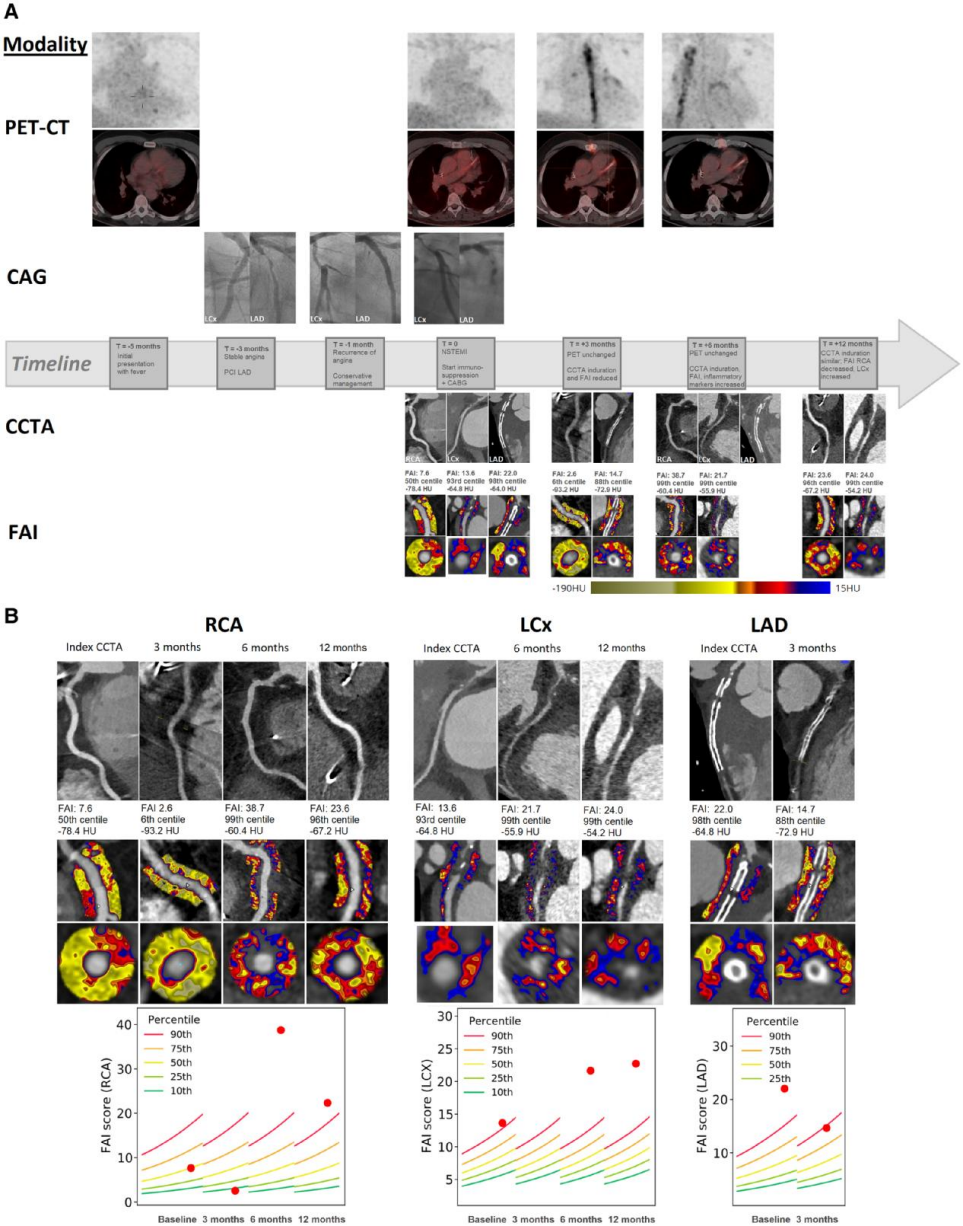
(*A*) Timeline of imaging acquired with corresponding clinical events and treatments received. (*B*) Fat attenuation index on index-coronary computed tomography angiography and at 3, 6, and 12 months after immunosuppression initiation for each individual coronary artery. RCA, right coronary artery; LCx, left circumflex artery; LAD, left anterior descending artery; PET, positron emission tomography; CCTA, coronary computed tomography angiography; FAI, fat attenuation index; NSTEMI, non-ST elevated myocardial infarction; PCI, percutaneous coronary intervention; CABG, coronary artery bypass grafting; HU, Hounsfield units. FAI images derived from CaRi Heart Analysis, Caristo Diagnostics.

At MI admission, FAI values on CCTA indicated severe inflammation in the LAD (22.0, 98th percentile) and LCx (13.6, 93rd percentile), while RCA-FAI was normal (7.6, 50th percentile) (*Figure 1*). After 3 months of immunosuppression, FAI decreased in both the LAD (14.7, 88th percentile) and RCA (2.6, 6th percentile), concordant with CCTA induration reduction, but discordant with PET, which remained similar. At 6 months, RCA FAI increased significantly (38.7, 99th percentile), and LCx FAI also increased compared to baseline (21.7, 99th percentile), indicating severe inflammation, concordant with increased induration on CCTA. This was consistent with CCTA and blood markers, while PET showed unchanged isolated LAD activity.

At 12 months, RCA and LCx FAI values still indicated substantial inflammation.

**Consent:** All authors were directly and majorly involved in medical care for this case and/or in imaging processing and interpretation. We can assure that all authors have contributed significantly to the manuscript and approved the submission of it. The patient has read and agrees with the contents of this manuscript and has provided written informed consent for publication. As this is a report of a single case, and no other data has been used, ethical approval has not been sought.

**Funding:** Caristo performed the CaRi Heart analyses required for this report as in-kind contribution. No other funding was acquired for this work. Caristo played no role in the interpretation of the results or the contents of this paper.

## Data Availability

The data generated for the assessment and treatment in this case have been presented in the manuscript. No new data were generated or analysed in support of this research outside of the data presented in the manuscript.
